# Expanding the Transcriptome of Head and Neck Squamous Cell Carcinoma Through Novel MicroRNA Discovery

**DOI:** 10.3389/fonc.2019.01305

**Published:** 2019-11-27

**Authors:** Leigha D. Rock, Brenda C. Minatel, Erin A. Marshall, Florian Guisier, Adam P. Sage, Mateus Camargo Barros-Filho, Greg L. Stewart, Cathie Garnis, Wan L. Lam

**Affiliations:** ^1^Department of Cancer Control Research, British Columbia Cancer Research Centre, Vancouver, BC, Canada; ^2^Faculty of Dentistry, University of British Columbia, Vancouver, BC, Canada; ^3^Department of Integrative Oncology, British Columbia Cancer Research Centre, Vancouver, BC, Canada; ^4^Faculty of Dentistry, Dalhousie University, Halifax, NS, Canada; ^5^Department of Pulmonology and CIC-CRB 1404, Rouen University Hospital, Rouen, France; ^6^International Research Center—A.C.Camargo Cancer Center, São Paulo, Brazil

**Keywords:** microRNAs, non-coding RNA, gene expression profiling, head and neck cancer, computational biology

## Abstract

Head and neck squamous cell carcinoma (HNSCC) has a poor survival rate mainly due to late stage diagnosis and recurrence. Despite genomic efforts to identify driver mutations and changes in protein-coding gene expression, developing effective diagnostic and prognostic biomarkers remains a priority to guide disease management and improve patient outcome. Recent reports of previously-unannotated microRNAs (miRNAs) from multiple somatic tissues have raised the possibility of HNSCC-specific miRNAs. In this study, we applied a customized *in-silico* analysis pipeline to identify novel miRNAs from raw small-RNA sequencing datasets from public repositories. We discovered 146 previously-unannotated sequences expressed in head and neck samples that share structural properties highly characteristic of miRNAs. The combined expression of the novel miRNAs revealed tissue and context-specific patterns. Furthermore, comparison of tumor with non-malignant tissue samples (*n* = 43 pairs) revealed 135 of these miRNAs as differentially expressed, most of which were overexpressed or exclusively found in tumor samples. Additionally, a subset of novel miRNAs was significantly associated with HPV infection status and patient outcome. A prognostic-model combining novel and known miRNA was developed (multivariate Cox regression analysis) leading to an improved death and relapse risk stratification (log rank *p* < 1e-7). The presence of these miRNAs was corroborated both in an independent dataset and by RT-qPCR analysis, supporting their potential involvement in HNSCC. In this study, we report the discovery of 146 novel miRNAs in head and neck tissues and demonstrate their potential biological significance and clinical relevance to head and neck cancer, providing a new resource for the study of HNSCC.

## Introduction

Head and neck squamous cell carcinoma (HNSCC) is the eighth most common cancer worldwide (1) and has a poor survival rate, mainly due to late stage diagnosis, and frequent disease recurrence (2). Despite advances in surgical techniques, chemotherapy, radiation therapy, and targeted therapy, the 5-years survival rate of patients remains at 50% (3). Hence there is a need to expand the repertoire of head and neck specific diagnostic and prognostic biomarkers. Furthermore, in order to improve patient outcome a better understanding of the genetic and epigenetic events associated with disease progression are needed.

MicroRNAs (miRNAs) are a class of single-stranded small non-coding RNAs (sncRNAs) ~21–23 nucleotides in length, which act as regulators of gene expression by binding to complementary sequences within mRNAs (4). A single miRNA transcript can act on multiple mRNA targets, and therefore, miRNAs are involved in many biological and pathological processes. In fact, miRNA dysregulation has been shown as a frequent and important event across all stages of cancer (5–8), as well as in many different cancer types (9–15). Their stability in biofluids and tissue biopsies presents opportunities for biomarker discovery (4, 16) and subsequently drug target detection (17–19). Among the dysregulated miRNAs in HNSCC, miR-21, miR-34, miR-93, miR-155, miR-196, and miR-211 are the most studied (20). Functional assays and target prediction have demonstrated that these miRNAs play important roles in regulation of cell proliferation, immune invasion, and resistance to cell death (21–24), corroborating their role as regulators in HNSCC (20, 25). Furthermore, miRNAs have demonstrated utility as biomarkers in the diagnosis and prognostication of HNSCC. For example, under-expression of let-7d and miR-205 are associated with poor survival in HNSCC (26), and circulating miR-142, miR-186, miR-195, miR-374b, and miR-574 have been shown to be promising markers for monitoring therapy in HNSCC patients (27).

While current miRNA repositories contain ~2,500 unique miRNA sequences, they are primarily comprised of those that are either conserved across several tissues or abundantly expressed, for the most part discounting lineage- and tissue-specific miRNAs (28). However, recent studies show that numerous miRNAs may be expressed only in specific tissues or contexts (29–33), and may have utility as clinical markers of disease (8, 34).

Mining of large-scale datasets using bioinformatic algorithms has become an important tool for expanding the current annotation of miRNA repositories and discovering these tissue/context-specific miRNAs, particularly due to the data's high coverage depth and sample size. The discovery of novel miRNAs not only provides a novel resource for the research community, but may also guide future clinical efforts on the design of new drug targets and disease biomarkers. Thus, we hypothesize the existence of previously-unannotated and tissue-specific miRNAs in head and neck samples, which may have been overlooked due to their tissue/context specificity. In this study, we use a large-scale analysis of high-throughput sequencing data to uncover these novel miRNAs and explore their relevance to HNSCC tumourigenesis.

## Materials and Methods

### Clinical Data Sets

A discovery cohort consisting of publicly available high-throughput raw small-RNA sequencing data from 523 tumors along with 43 paired non-malignant samples was retrieved from The Cancer Genome Atlas (TCGA) on the cgHUB data repository (dbgap Project ID: 6208), available at: https://cancergenome.nih.gov/ (accessed October 2018). Clinical information on the cases, summarized in Table 1, was obtained from the University of California Santa Cruz Xena Browser, available at: https://xenabrowser.net/ (accessed August 2018). HPV status was obtained from the Cancer Genome Atlas Network (35).

**Table 1:** Clinicopathological information of the HNSCC patients from TCGA^*^.

**Clinical feature**		**Total (%)^$^**
Histology	Malignant	523
Anatomical Site	Oral cavity	316 (60.4)
	Pharynx	90 (17.2)
	Larynx	117(22.4)
Age^†^	Range	19–90
	Median	61
Gender	Male	382 (73.0)
	Female	141 (27.0)
Smoking status	Never smoker	121 (23.1)
	Former smoker	211 (40.3)
	Current smoker	176 (33.7)
	Not determined	15 (2.9)
Disease stage	I	21 (4.0)
	II	97 (18.5)
	III	105 (20.1)
	IVA, IVB, and IVC	286 (54.7)
	Not determined	14 (2.7)
HPV status^#^	Positive	73 (14.0)
	Negative	40 (7.6)
	Not determined	410 (78.4)

* *Information retrieved August 2018 from UCSC Xena (https://xenabrowser.net)*.

$ *Column percentage*.

† *Age data missing for one patient*.

# *Determined by p16 testing*.

Publicly available small-RNA sequencing data from an independent cohort (*n* = 20) of oral squamous cell carcinoma samples were obtained from the Gene Expression Omnibus (GEO) repository (Accession GSE52663) (36).

Validation was carried out using formalin-fixed paraffin-embedded (FFPE) tissue from 25 oral squamous cell carcinoma (OSCC) tumors and 5 non-malignant oral tissue samples.

### Data Processing and Novel MicroRNA Discovery

**Figure 1:**
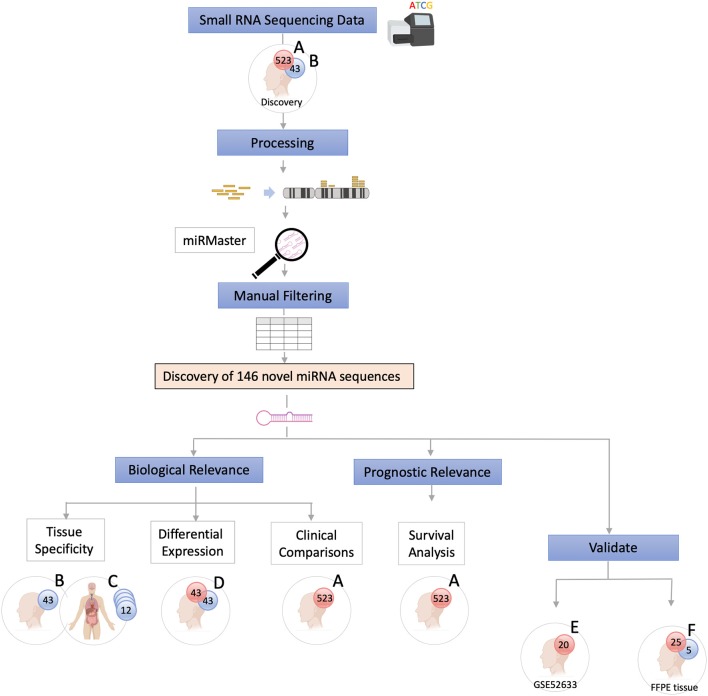
Study Flow Chart. High throughput small RNA-sequencing data from head and neck squamous cell carcinoma (HNSCC) (*n* = 523, dataset A) and matched non-malignant tissue (*n* = 43, dataset B) were obtained from The Cancer Genome Atlas (TCGA). Raw sequence data (BAM files) were converted into unaligned reads (FASTQ) and inputted into miRMaster for miRNA detection and quantification. A threshold criteria of ≥1 read per million (RPM) in ≥10% of samples per group was employed. To determine whether these novel sequences have potential biological relevance group comparison and association analyses were performed. Tissue specificity of the novel candidate sequences was assessed by comparing non-malignant samples (dataset B) with those from 12 other non-malignant tissue types from TCGA Pan-Cancer Atlas (dataset C) using non-linear t-Distributed Stochastic Neighbor Embedding. Differentially expressed novel miRNAs were detected by comparing tumor and matched non-malignant samples (dataset D). Clinicopathological features of the novel miRNA transcripts (*n* = 130) that were found to be expressed exclusively in tumor samples (dataset A) were compared. Survival analysis was performed to further characterize the novel sequences. Cox regression analysis showed that candidate novel miRNA sequences behave similarly to known miRNAs and may have prognostic value. Validation was performed on an independent dataset (Gene Expression Omnibus GSE52633) (dataset E) and by performing RT-qPCR of the most relevant miRNA candidates in formalin-fixed paraffin-embedded (FFPE) tissues (dataset F).

The data were analyzed using a customized *in-silico* analysis pipeline. The study design is summarized in Figure 1, and the data subsets used for the step-wise comparisons that were conducted are summarized in Table 2.

**Table 2:** Description of clinical data sets.

**Data set**	**Description of samples**
A	HNSCC samples obtained from TCGA (*n =* 523)
B	Non-malignant head and neck samples obtained from TCGA (*n =* 43)
C	Non-malignant samples from different organs^*^ from TCGA Pan-Cancer Atlas
D	Matched HNSCC and non-malignant samples from TCGA (*n =* 43 pairs)
E	OSCC from the GEO (GSE52633) (*n =* 20)
F	FFPE OSCC tissue (*n =* 25) and FFPE non-malignant tissue from the buccal mucosa (*n =* 5)
**Analyses**	
A and B	MiRNA discovery
B and C	Tissue specificity^*^ of novel miRNAs
D	Differential expression between non-malignant samples and HNSCC
A	Association of miRNAs with clinical features
A	Survival analysis
E	Detection of novel miRNAs in an independent cohort
F	Experimental validation of most relevant miRNA by RT-qPCR in FFPE tissues

*HNSCC, head and neck squamous cell carcinoma; TCGA, The Cancer Genome Atlas; OSCC, oral squamous cell carcinoma; FFPE, formalin-fixed paraffin-embedded*.

* *bile duct (n = 9), bladder (n = 19), brain (n = 5), cervix (n = 3), colon (n = 9), kidney (n = 71), liver (n = 47), lung (n = 91), pancreas (n = 4), prostate (n = 52), stomach (n = 45), and thyroid (n = 59)*.

Raw sequence data from both HNSCC tumors and non-malignant head and neck tissue samples (Table 2, datasets A and B) obtained from TCGA in the form of BAM files were converted into unaligned (FASTQ) files using Partek Flow^®^ (http://www.partek.com/partek-flow/). FASTQ files were then analyzed for novel miRNA expression using the online analysis platform miRMaster (https://ccb-compute.cs.uni-saarland.de/mirmaster) (accessed October 2018). This platform predicts novel miRNAs based on the miRDeep2 algorithm, a well-established novel miRNA discovery tool which identifies miRNA-like configurations by considering relative free-energy and the probability of random folding (37). Default parameters were used to perform quality filtering and read collapsing. The adapters were trimmed (Illumina TruSeq small RNA 3p), followed by the alignment of the reads to the hg38 build of the human genome (38). Sequences previously annotated in miRBase v.22 were excluded. The list of candidate novel miRNA transcripts was then further curated to include only sequences with a detectable expression of ≥1 read per million (RPM) in at least 10% of samples, for each group. Those miRNA candidates that remained after filtering were considered putative novel miRNAs.

To verify their designation as true miRNA sequences, we assessed whether these novel miRNA candidates shared structural properties and sequence features with known miRNA sequences. Nucleotide composition of the seed sequence and guanine-cytosine (GC) content were compared between the novel candidates and currently-annotated miRNAs, as well as their distribution across the genome.

### Group Comparison and Association Studies

To determine the tissue-specificity of these novel miRNA candidates, normalized expression levels of the 146 candidate novel miRNA sequences from the non-malignant head and neck tissues (Table 2, datasets B and D) were queried against non-malignant samples from 12 different organ sites from TCGA Pan-Cancer Atlas using non-linear t-Distributed Stochastic Neighbor Embedding (t-SNE) dimensionality reduction. The tissues investigated included bile duct (*n* = 9), bladder (*n* = 19), brai*n* (*n* = 5), cervix (*n* = 3), colo*n* (*n* = 9), kidney (*n* = 71), liver (*n* = 47), lung (*n* = 91), pancreas (*n* = 4), prostate (*n* = 52), stomach (*n* = 45), thyroid (*n* = 59) and head & neck (*n* = 43).

To assess their involvement in HNSCC development, we sought to determine whether these novel transcripts are dysregulated in corresponding tumor samples.

An unsupervised hierarchal clustering analysis (Pearson correlation and complete linkage) was performed including novel miRNAs present in both tumor and non-malignant sample groups (Table 2, dataset D). Paired sample *t*-test (Benjamini-Hochberg [BH] adjusted *p* < 0.05 and fold change [FC] > 1.5) was applied to compare the novel miRNA expression between malignant and non-malignant samples (*n* = 43 pairs).

Clinical-pathological associations, examining anatomical site (oral cavity, pharynx, and larynx), smoking status (lifelong non-smoker versus continuing smoker) and HPV status (negative vs. positive), were observed for the novel miRNAs (*n* = 130) expressed exclusively in tumor samples (Table 2, dataset A) (*t*-test BH adjusted *p* < 0.05 and FC > 1.5).

To explore a potential prognostic relevance of the sequences discovered, the miRNA expression was associated with overall (OS) and recurrence-free survival (RFS) using the TCGA tumor samples (Table 2, dataset A). MicroRNAs associated with survival (*p* < 0.01) in a univariate log-rank test were included in a multivariate Cox proportional hazard model.

### Target Prediction and Pathway Enrichment

To investigate the possible genes targeted by our recently discovered miRNAs and their biological roles, we performed target prediction and pathway enrichment analysis. Target prediction was performed using the miRanda v 3.3a algorithm, against all human genes 3′ UTR sequences acquired from Ensembl through Biomart tool (https://www.ensembl.org/) (39). The prediction algorithm was executed using strict alignment, alignment score ≥180 and energy threshold ≤ -20 kcal/mol parametrizations. Next, to gain further functional insights into the pathways these targets may be involved, we submitted the gene symbols identified to a comprehensive pathway enrichment analysis using pathDIP, which includes 15 distinct pathways resources (Extended pathway associations. Experimental plus orthologs plus FpClass – High Confidence; Minimum confidence level for predicted associations: 0.99) (40).

### Confirmation Using an Independent Cohort

Publicly available small-RNA sequencing data from a second cohort (*n* = 20) (Table 2, dataset E) of oral squamous cell carcinoma (OSCC) tissue samples were downloaded from GEO (Accession GSE52663) (36). SRA files were converted to FASTQ and mapped to human genome build 38 using the STAR aligner in Partek Flow^®^ (41). Novel miRNA candidates were then quantified by their genomic loci. Expression values were averaged to create an average expression value per sample. A detection threshold ≥10 reads across the averaged samples was employed.

### Confirmation by RT-qPCR

To further confirm the presence of these miRNAs in HNSCC, we selected five of the most highly-expressed HNnov-miRNAs and confirmed their expression by PCR in an independent cohort of OSCC. Formalin-fixed paraffin-embedded (FFPE) tissue blocks (*n* = 25 OSCC and 5 normal oral tissue from the buccal mucosa) (Table 2, dataset F) were obtained from the British Columbia Oral Biopsy Service using written informed consent and a study protocol approved by the University of British Columbia—BC Cancer Research Ethics Board. Five 10 μm sections were cut from each block, and immediately placed into clean 1.5 mL microtubes. Deparaffinization was performed in xylene, and extraction was performed using the miRNeasy FFPE kit (QIAGEN, Hilden Germany) following manufacturer's guidelines.

Custom reverse-transcription and PCR primers were designed using the Custom TaqMan^®^ Small RNA Assay Design Tool from Thermo Fisher. Primers were designed specific to the mature miRNA sequences for five of the highest-expressing novel HNnov-miRNAs, including HNnov-miR-59-5p (UGAGUUCUGGGCUGUAGUGUGCU), HNnov-miR-3-5p (AAUUACAGAUUGUCUCAGAGA), HNnov-miR-45-5p (GGGGGUGUAGCUCAGUGGUAGA), HNnov-miR-19-5p (CCCUGAUGAGCUUGACUCUAG), and HNnov-miR-48-3p (AAGUUUCUCUGAACGUGUAGAGC), according to Table S1. Reverse transcription of miRNA species was performed using the TaqMan™ MicroRNA Reverse Transcription Kit (Applied Biosystems™, Cat#4366596) and RT-qPCR in TaqMan™ Universal Master Mix II, with UNG (Applied Biosystems™, Cat#4440044) according to protocols established by the manufacturer. RT-qPCR was performed in an Applied Biosystems^®^ 7500 Real-Time PCR System, and expression of mature miRNA transcripts in tumors was calculated in reference to normal oral epithelium using the 2^(−ΔΔ*Ct*)^ method and normalized to U6 (TaqMan Cat#4427975, Assay ID: 001973).

## Results

### Discovery of Novel miRNA Sequences in Head and Neck Samples

**Figure 2:**
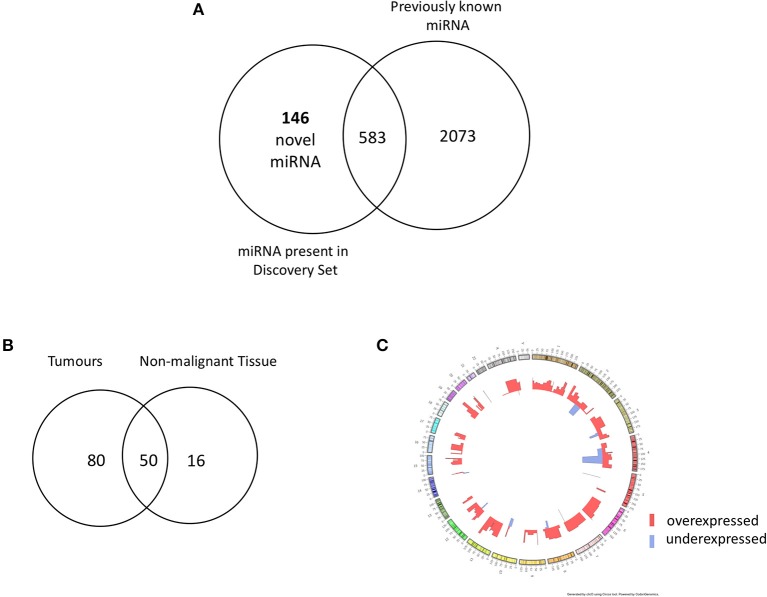
**(A)** Venn diagram summarizing the relative proportion of novel vs. previously identified miRNAs expressed to the same levels in the TCGA cohort compared to the current annotation of miRNA repositories. An addition of 146 novel miRNAs to 583 previously annotated sequences expressed to the same d level in the TCGA increases the transcriptome head and neck tissues substantially. **(B)** Venn diagram of novel miRNAs identified in head and neck squamous cell carcinoma tumor tissue (*n* = 523) and non-malignant (*n* = 43) tissue. Our results revealed 146 novel miRNA candidates; 80 and 16 were observed exclusively in non- malignant and tumor tissues, respectively, with 50 miRNA candidates detected in both groups. **(C)** Circos plot displaying the genomic localization of the novel miRNAs. The outermost circle displays the human autosomal chromosomes, and the inner layers show the expression fold changes (logged) of the novel miRNAs in head and neck squamous cell carcinoma tumors in relation to matched non-malignant tissue [created by ClicO FS: An interactive web-based service of Circos (42)].

In order to identify novel miRNAs in HNSCC non-malignant and tumor tissues, we submitted the raw HNSCC sncRNA sequence data from TCGA (Table 2, datasets A and B) to the online platform miRMaster and applied a miRNA-discovery algorithm as described in Materials and Methods. This initial analysis resulted in a list of miRNA candidates that were curated to exclude sequences highly homologous to those previously annotated in miRBase v.22. After curation, 146 previously unannotated miRNAs were identified (Table S1). These novel miRNA sequences are herein referred to as HNnov-miRs. The discovery of these 146 miRNAs represents a 5.5% increase to the total number of 2,656 currently-annotated miRNAs quantified by miRMaster, and an outstanding increase of 25% to the 583 currently-annotated miRNAs that were also found to be expressed at our threshold levels (1 RPM in 10% of the samples) in the TCGA HNSCC cohort (Figure 2A). Like currently-annotated miRNAs, the HNnov-miRs where shown to be widely distributed across the genome (Figure 2C). Additionally, they were found to have similar overall molecular features compared to annotated miRNAs, further supporting their identity as miRNA sequences (Table S1).

### Tissue- and Context-Specific Expression Patterns of the Novel miRNAs

**Figure 3:**
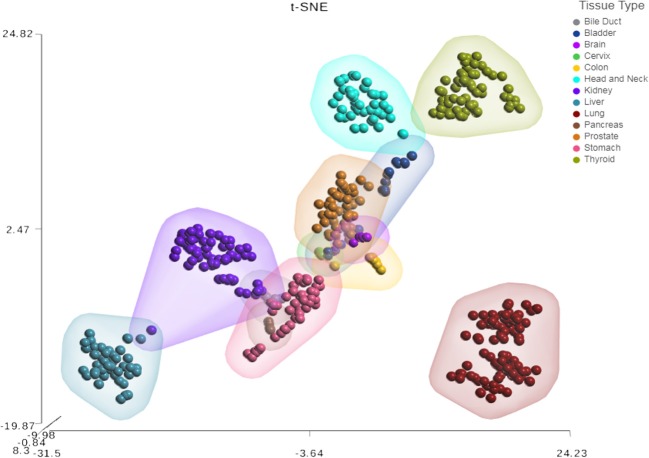
Tissue-specific expression patterns of unannotated miRNA transcripts. t-Distributed Stochastic Neighbor Embedding (t-SNE) analysis T-SNE shows tissue specificity of head and neck non-malignant tissue compared to other non-malignant tissue from The Cancer Genome Atlas (TCGA); bile duct (*n* = 9), bladder (*n* = 19), brain (*n* = 5), cervix (*n* = 3), colon (*n* = 9), were compared to head & neck (*n* = 43), kidney (*n* = 71), liver (*n* = 47), lung (*n* = 91), pancreas (*n* = 4), prostate (*n* = 52), stomach (*n* = 45), and thyroid (*n* = 59).

Next, we sought to investigate the tissue-specificity of the HNnov-miRs by comparing their combined expression patterns in head and neck against other tissue types. This analysis showed that the HNnov-miRs are indeed head and neck-specific and their combined expression patterns were able to clearly distinguish non-malignant head and neck samples from other types of non-malignant tissue (bile duct, bladder, brain, cervix, colon, kidney, liver, lung, pancreas, prostate, stomach, and thyroid), as evidenced by t-Distributed Stochastic Neighbor Embedding (t-SNE) analysis (Figure 3). This tissue-specific nature highlights their potential relevance to head and neck biology.

### Differential Expression in HNSCC Tumor and Non-malignant Head and Neck Tissue

**Figure 4:**
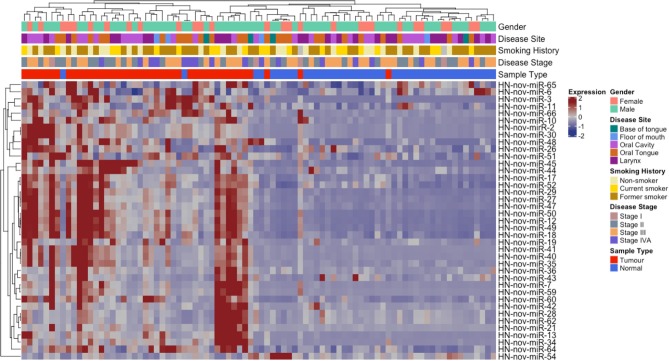
Unsupervised hierarchal clustering analysis comprising 39 HNnov-miR expressed in both tumors and non-malignant tissue. The dendogram shows two clusters, the first enriched by non-neoplastic samples (novel miRNA expression predominantly low) and the second by tumor samples (novel miRNA expression predominantly high). Heatmap annotation bars show some of the clinical parameters associated with each tissue sample, including gender, disease site and stage, smoking history, and tissue type.

From our curated list of 146 HNnov-miRs, a total of 16 HNnov-miR sequences were exclusively expressed in non-malignant samples, 80 in tumors only, and 50 shared between both sample types (Figure 2B, Table S2). Of the 50 HNnov-miRs detected in both matched tumor and non-malignant tissue samples (*n* = 43 pairs), 39 were differentially expressed (BH-*p* < 0.05). Most sequences (*n* = 38) were found to be significantly over-expressed in HNSCC, while only one was under-expressed in tumors compared to non-malignant tissue (Table S2). Hierarchical clustering analysis of the HNnov-miRs detected in both tumor and matched non-malignant tissue samples demonstrated a clear difference in expression patterns between the two groups (Figure 4), which highlights that the HNnov-miRs are not only tissue-specific but also context-specific.

To further explore the role of these 39 HNnov-miRNAs found to be significantly over-expressed in HNSCC, we performed target prediction analysis. This analysis revealed a total of 10,221 possible unique protein-coding gene targets (Table S2), in which 3,273 were targeted by at least 10% of the 39 miRNAs. We also performed pathway enrichment analysis on the 10,221 gene targets to investigate the biological pathways they may be involved and reported the top 20 enriched pathways (Table S6). In this analysis, none of the pathways were found to be significantly enriched after Benjamini-Hochberg correction, however it suggests the target genes to be involved mainly with interleukin signaling.

We also investigated if HNnov-miRs expression patterns differed according to different clinical parameters. Expression patterns of the novel miRNAs did not differ significantly between oral cavity and pharynx/larynx subsites. Likewise, expression between smokers and non-smokers did not differ significantly. Interestingly, three of the predicted novel miRNAs (HNnov-miR-2, HNnov-miR-30, and HNnov-miR-125) were significantly associated with HPV status (BH-*p* < 0.05 and fold change>1.5), where their downregulation was associated with the presence of HPV infection (Table S3).

### Potential Prognostic Relevance of the Novel miRNAs

The prognostic impact of novel and known miRNAs was assessed in the TCGA cohort (*n* = 523) (dataset A in Table 2). Three predicted novel miRNAs were significantly associated with overall survival (OS; HNnov-miR-104, HNnov-miR-120, and HNnov-miR-136) and three were significantly associated with recurrence free survival (RFS; HNnov-miR-3, HNnov-miR-87, and HNnov-miR-135) in univariate analyses (Table S4, Figure S1). In a multivariate Cox proportional hazard model including both novel and known miRNAs, one novel miRNA remained independently associated with OS (HNnov-miR-120), and two with RFS (HNnov-miR-3 and HNnov-miR-135). We then established scores for OS and RFS using either known miRNAs alone or both novel and known miRNAs. Scores using novel and known miRNAs were more powerful in the segregation of patients within prognostic groups (Table S4, Figure S2).

### Confirmation of the Novel miRNAs in an Independent Cohort

To confirm the existence of our novel miRNAs, we also investigated their presence in an additional RNA-sequencing dataset using the same analysis and filtering criteria performed in our discovery cohort. In the validation dataset (Table 2, dataset E), 102 of the 146 HNnov-miRs were detected (Table S5, Figure S3), including all three of the HNnov-miRs that were found to be overexpressed in HPV negative samples and all six of the HNnov-miRs that were associated with OS or RFS.

### Validation by RT-qPCR

For this verification, we found that, compared to normal tissues, the 5 miRNA selected were all more highly expressed in OSCC, confirming not only their existence within the tumor, but their importance to tumor biology (Figure S4).

## Discussion

In this study, we report a comprehensive analysis of undiscovered miRNAs that has led to the expansion of the head and neck transcriptome. By analyzing raw small-RNA sequencing data for both quantity and secondary RNA structure, we discovered 146 HNnov-miRs previously undescribed in head and neck tissues. Our characterization of these novel transcripts has revealed not only their tissue-specific nature and their context-specific expression patterns relevance to head and neck cancer biology, but also their diagnostic and prognostic potential.

The current annotation of the human miRNA transcriptome mainly contains miRNA sequences that are abundant and conserved. Therefore, cell lineage- and tissue-specific miRNAs, especially those that are less abundant, may not be included in current miRBase annotations (29). This study, like several recent studies of other organs, has shown that re-analyses of high-throughput sequencing data, can lead to large-scale discoveries of novel miRNAs that are expressed in a tissue-specific manner, thus expanding the human miRNA transcriptome (29–33).

In order to validate the expression of the 146 HNnov-miRs, we analyzed an independent dataset of HNSCC (*n* = 20). High throughput sequencing data of small-RNAs are scarce, and despite the limited sample size of this validation set, 102 of our HNnov-miRNAs were detected in this independent cohort. To provide an additional layer of verification, experimental validation of the miRNAs was carried out by performing RT-qPCR of the most relevant miRNA candidates in OSCC tissues, thereby strengthening the position that these novel miRNAs may serve as a new resource for the exploration of head and neck cancer specific transcripts in future investigations.

Interestingly, our study did not show a difference in expression pattern of HNnov-miRNA between HNSCC tumors from smokers and non-smokers. These observations are sustained by similar studies. Kolokythas et al. have reported similar miRNA expression in oral squamous cell carcinoma in never-smokers and ever-smokers (43). Similarly, a study that looked at genome wide analysis in 30 oral potentially malignant lesions that progressed to cancer and a study that examined loss of heterozygosity at 9p, 17p, and 4q in 455 lesions with oral epithelial dysplasia showed similar genetic alterations between smokers and nonsmokers (44, 45). However, Irimie et al. have reported that the overall variation in gene expression profiles was different for patients who smoked compared to those who have never smoked. The interaction between genetics and exposure to non-tobacco environmental carcinogens complicates the identification of a single effect, such as smoking, related to HNSCC.

**Figure 5:**
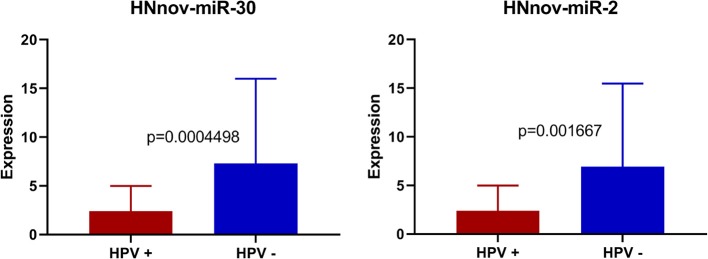
Expression of HNnov-miR-2 and HNnov-miR-30 is significantly associated with negative HPV status in tumors (Mann Whitney *U*-test).

Our results showed that three of the predicted novel miRNAs (HNnov-miR-2, HNnov-miR-30, and HNnov-miR-125) were significantly associated with HPV status. Interestingly, all of these novel genes map to chromosome 12, and both HNnov-miR-2 and HNnov-miR30 lie within the genes *KRT6C* and *KRT6B*, respectively. This is interesting as both KRT6C, and B, have previously described to have roles in various cancers, and are included in a gene signature separating lung adenocarcinoma, from lung squamous cell carcinoma (46, 47). Further, we also find expression of these genes to be associated with HPV status. Additional studies will be needed to determine if these novel miRNAs work in conjunction with, or have specific functions independent of these cancer associated protein coding genes (Figure 5).

The potential utility of the HNnov-miRNAs is highlighted by our observations that a subset of these transcripts is significantly associated with patient outcome (Figure S1), and that combining novel and known miRNAs improved the prognostic signature (Figure S2). The expression of HNnov-miR-120, HNnov-miR-3 and HNnov-miR-135 have prognostic relevance regarding recurrence-free and overall survival in patients with HNSCC and may improve the current prognostic risk stratification of HNSCC.

Here, to investigate if the unannotated miRNAs discovered in head and neck tissue were tissue specific, we assessed a number of non-malignant datasets generated by TCGA, including some cohorts with low sample numbers. In general, the more samples of a tissue type analyzed, the greater likelihood of discovering additional unannotated miRNA transcripts, especially those with non-constitutive or low expression levels. Therefore, a caveat of this analysis is that some of the HNnov-miRs may have not been detected in the additional tissues analyzed because of the low sample numbers, particularly in the cohorts such as brain and cervix. However, it can indicate that they if present in these other tissues, they display different expression levels and their combined patter of in head and neck are quite tissue specific. While this study represents the first-generation analysis of these unannotated miRNAs, and focuses on head and neck tissue, future studies with additional samples will be needed to comprehensively catalog these species across human tissues.

Although we cannot weigh the HNnov-miRNAs newly discovered in this study against literature, we can assess whether the expression and function of the known miRNA observed within our custom pipeline are consistent with what is found in the literature. Our findings are consistent with a systematic review of 21 studies by Jamali et al. which indicated that overexpression of miR-18a, miR-19a, miR-21, miR-134a, and miR-155, miR-181a, miR-210, were associated with poor survival, and that significantly decreased expression of let-7d, let-7g, miR-17, miR-34a, and miR-125b, miR-126a, miR-153, miR-200c, miR-203, miR-205, miR 218, miR-363, miR-375, miR-491-p5, miR-451, were associated with poor prognosis (48). In our study, we analyzed miRNA expression in the TCGA dataset (*n* = 523, dataset A), and found that among the abovementioned miRNAs, miR-134a, miR-153, miR-200c, miR-205, and miR-125b were significantly associated with overall survival in univariate analysis. After controlling for heterogeneity, Jamali's fixed model meta-analysis indicated that a significantly increased expression of miR-21 is associated with poor survival (Pooled HR = 1.57–95% CI: 1.22–2.02, *P* < 0.05) (48). In multivariate analysis, we found that only miR-205 remained significantly associated with overall survival. These findings add weight to the relevance and legitimacy of the novel miRNA discovered within our pipeline.

In conclusion, annotated miRNAs represent only a fraction of all the miRNAs encoded by the human genome. Here we identified 146 HNnov-miRs expressed in head and neck tissues with potential relevance to HNSCC biology, as well as diagnostic and prognostic potential. While our study was performed on a predictive platform and mainly relied on small-RNA sequencing data, the validation of 5 of these novel miRNAs by RT-qPCR supported their existence. Likewise, to understand their biological role and potential clinical utility, further functional assays will be required. An important next step would be to query the presence of these HNnov-miRNAs in liquid biopsies, such as serum samples. Here, we expand the current repertoire of head and neck miRNAs and provide an important new resource for the exploration of organ and disease specific transcripts that may guide future discoveries in head and neck cancers.

## Data Availability Statement

All data analyzed in this study are publicly available: TCGA consortium/NIH GDC (https://gdc.cancer.gov/); and GEO database accession number: GSE52663.

## Ethics Statement

The studies involving human participants were reviewed and approved by University of British Columbia Research Ethics Board. The patients/participants provided their written informed consent to participate in this study.

## Author Contributions

LR and BM were responsible for the project design. LR, BM, EM, FG, AS, MB-F, and GS contributed to data acquisition, data analysis, interpretation of results, and manuscript preparation. CG and WL were principle investigators of this project. All authors have read, edited and approved the final manuscript, and agree to be accountable for the content of the work.

### Conflict of Interest

The authors declare that the research was conducted in the absence of any commercial or financial relationships that could be construed as a potential conflict of interest.
