# Supplementary figures and images for: Expanding the Transcriptome of Head and Neck Squamous Cell Carcinoma Through Novel MicroRNA Discovery
Source: Front Oncol. 2019 Nov 27;9:1305. doi: 10.3389/fonc.2019.01305 (PMC6890850; doi:10.3389/fonc.2019.01305)

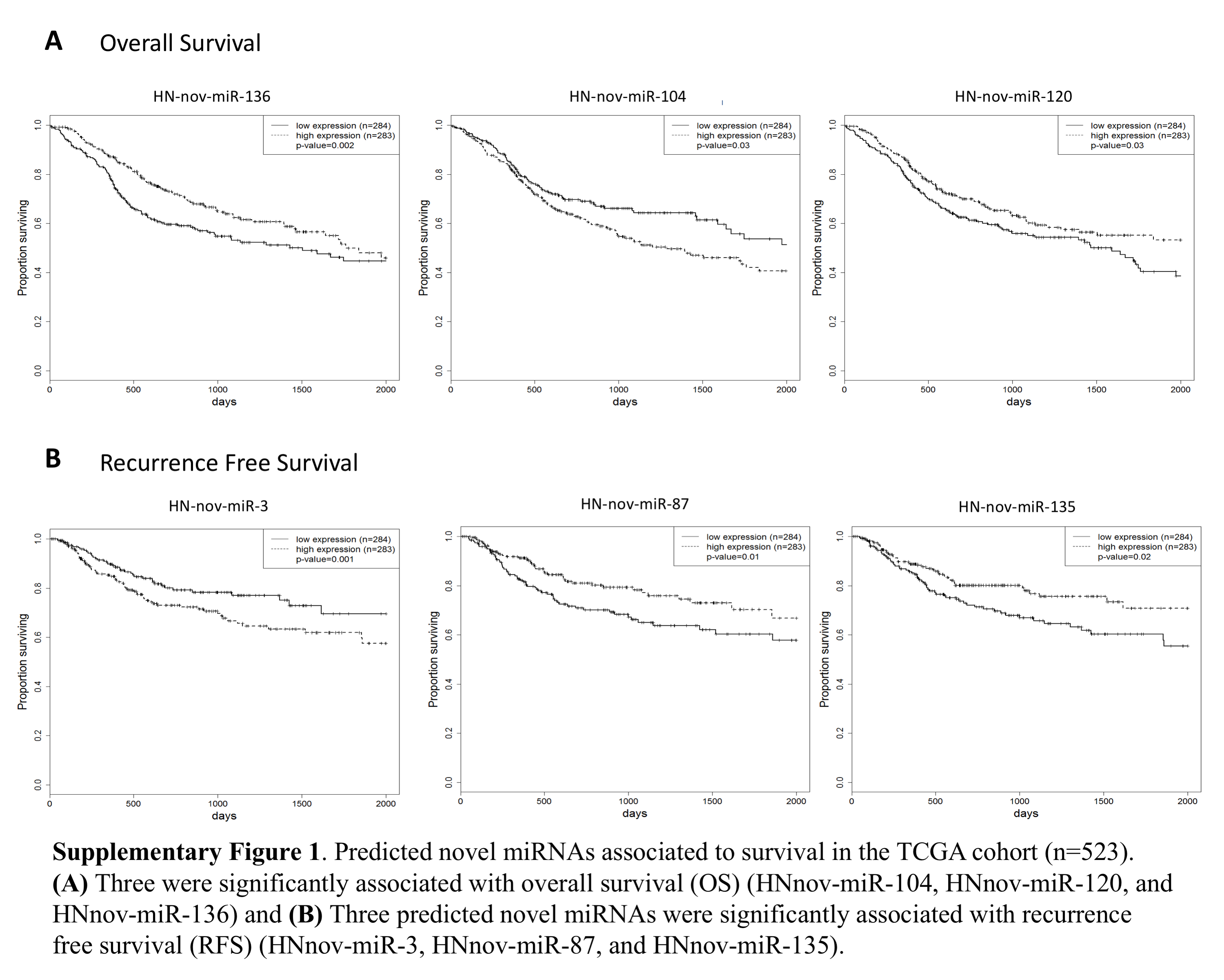

Supplement: Supplementary file 2 [file Image_1.TIFF]

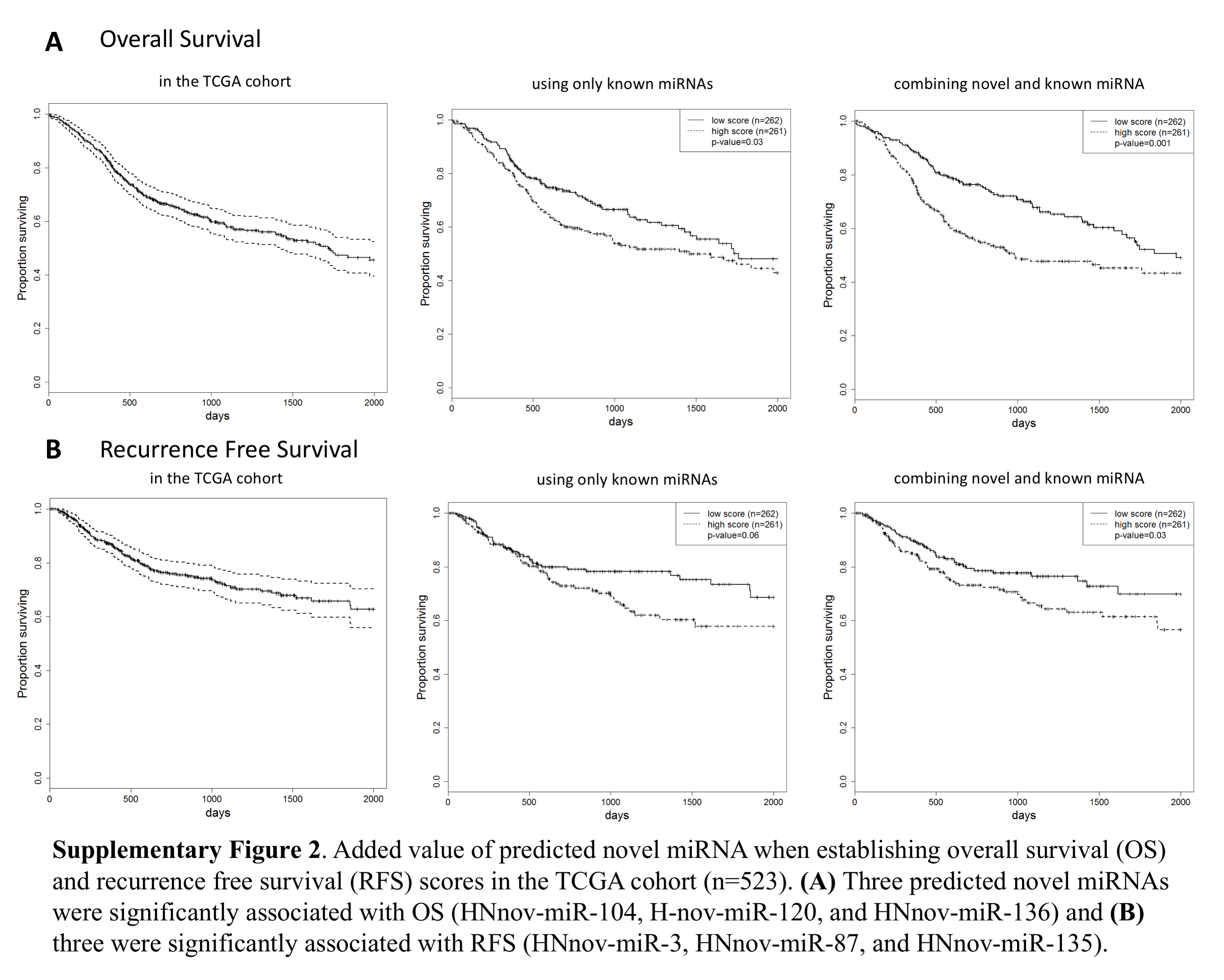

Supplement: Supplementary file 3 [file Image_2.TIFF]

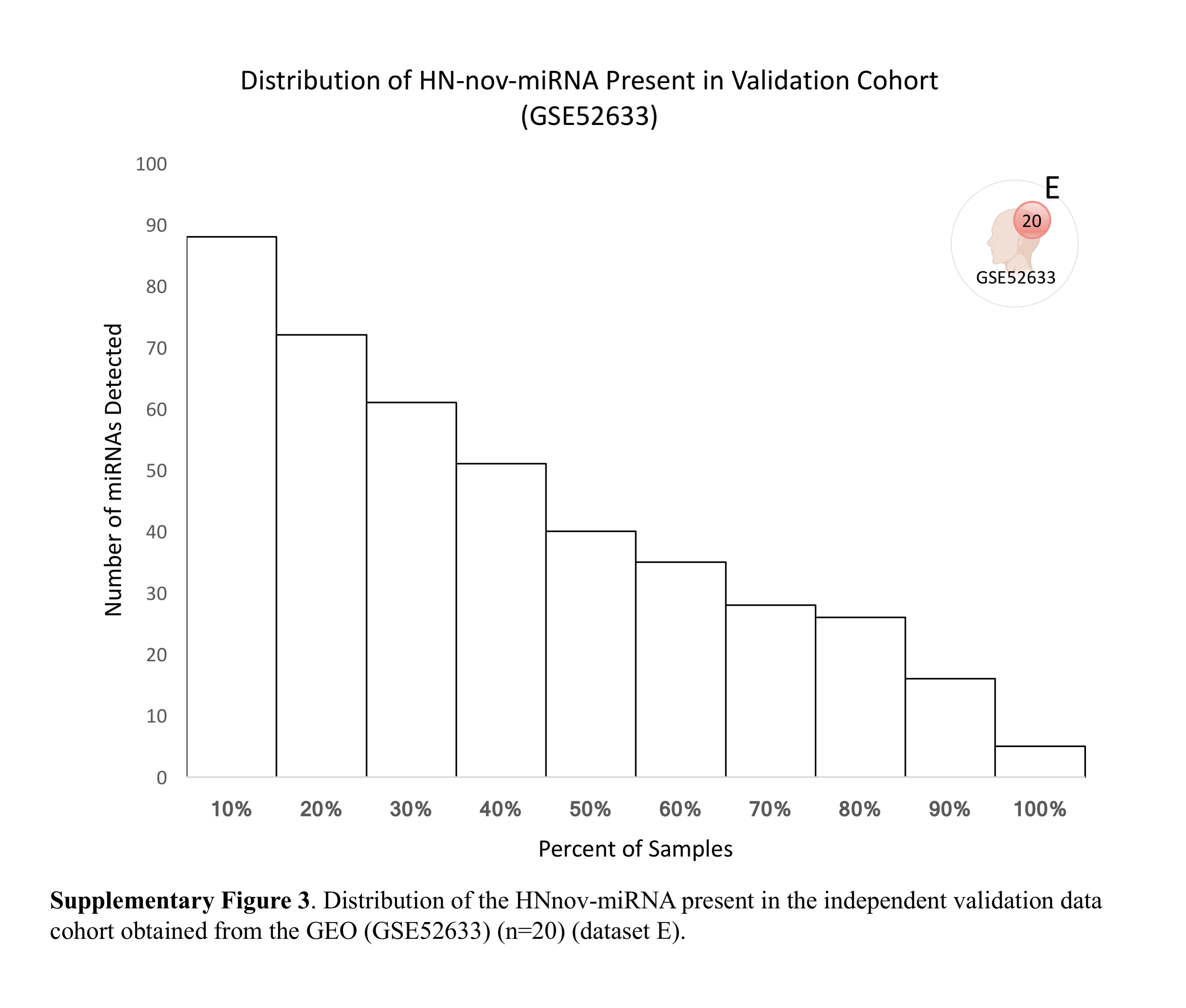

Supplement: Supplementary file 4 [file Image_3.TIFF]

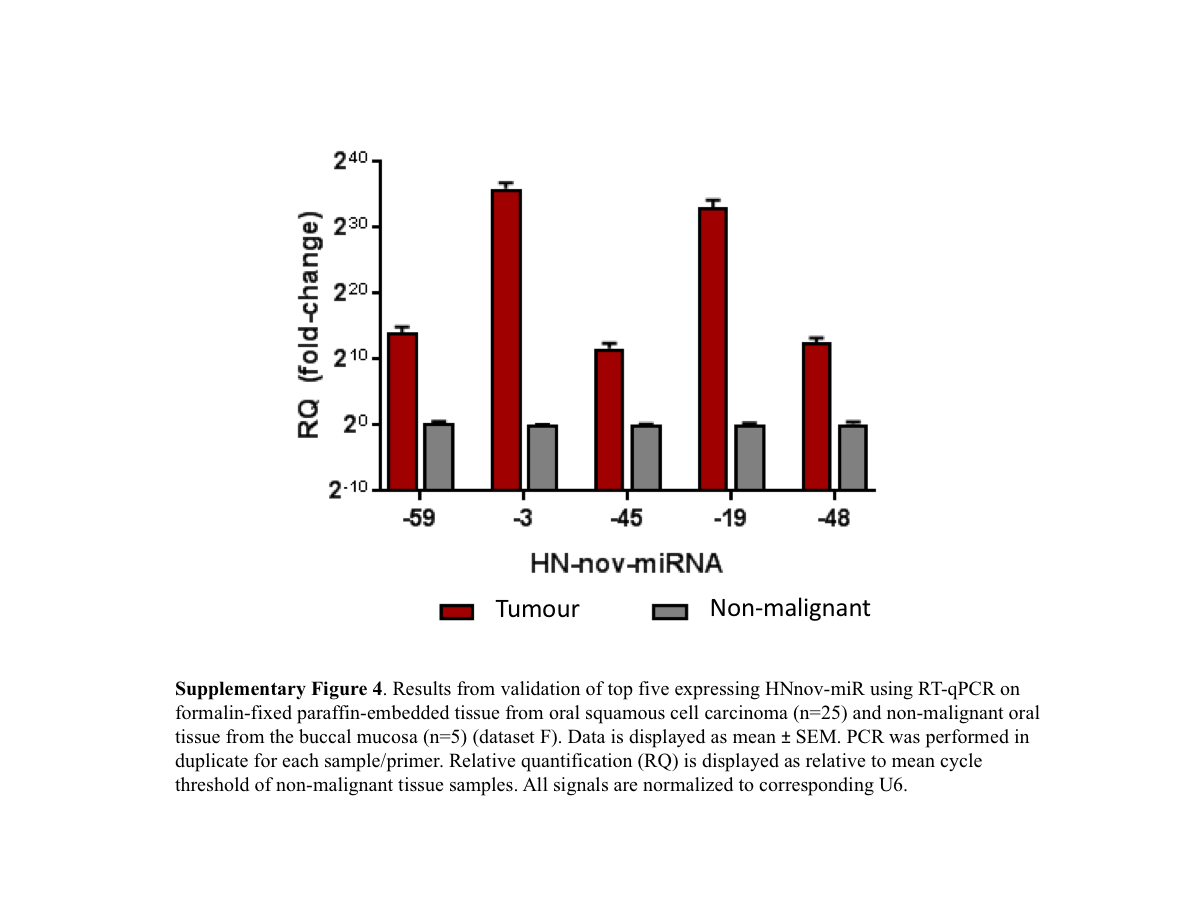

Supplement: Supplementary file 5 [file Image_4.tiff]
